# HIV–Tuberculosis Co-Infection Among Immigrants and Refugees in the United States: An Integrative Review

**DOI:** 10.3390/ijerph23080987

**Published:** 2026-07-29

**Authors:** Philip Ogah Abutu, Marcia Y. Shade, Susan Barnason, Chad Abresch, Nada Fadul, Regina Idoate, Keyonna M. King

**Affiliations:** 1 Health Promotion and Disease Prevention Research, University of Nebraska Medical Center, Omaha, NE 68198, USA; cabresch@unmc.edu (C.A.); regina.robbins@unmc.edu (R.I.); keyonna.king@unmc.edu (K.M.K.); 2 College of Nursing, University of Nebraska Medical Center, Omaha, NE 68198, USA; marcia.shade@unmc.edu (M.Y.S.); sbarnaso@unmc.edu (S.B.); 3 College of Medicine, University of Nebraska Medical Center, Omaha, NE 68198, USA; nada.fadul@unmc.edu

**Keywords:** HIV, human immunodeficiency virus, TB, tuberculosis immigrant, refugee

## Abstract

**Highlights:**

**Public health relevance—How does this work relate to a public health issue?**

- HIV-tuberculosis (HIV-TB) co-infection remains a major public health challenge because tuberculosis is the leading cause of death among people living with HIV worldwide, and immigrants and refugees account for the majority of tuberculosis cases in the United States.
- This review highlights how cultural stigma, socioeconomic marginalization, and psychological stressors including fear of deportation, language barriers, and trauma limit timely diagnosis, treatment adherence, and engagement with HIV and TB care among immigrant and refugee populations in the United States.

**Public health significance—Why is this work of significance to public health?**

- By synthesizing evidence from multiple study designs, this review provides a comprehensive understanding of the cultural, social, and psychological determinants shaping HIV-TB co-infection care among immigrants and refugees in the United States.
- The study advances current knowledge by demonstrating that barriers to HIV-TB care are not solely biomedical but are strongly influenced by structural and social determinants of health, highlighting the need for multidisciplinary and community-informed interventions to reduce health disparities.

**Public health implications—What are the key implications for practitioners, policymakers, and researchers in public health?**

- Public health practitioners should implement culturally and linguistically tailored HIV and TB screening, education, and treatment programs that address stigma, misinformation, and barriers related to migration and language.
- Policymakers should strengthen equitable healthcare access for immigrant and refugee populations by supporting community-based services, culturally competent care models, and policies that reduce structural barriers such as lack of insurance and fear of immigration enforcement.

**Abstract:**

HIV-tuberculosis (HIV-TB) co-infection remains a leading cause of morbidity and mortality among people living with HIV and disproportionately affects immigrant and refugee communities in the United States. While antiretroviral therapy has improved HIV outcomes, cultural, social, and psychological barriers hinder timely diagnosis and sustained HIV-TB co-infection care in these populations. This integrative review aims to synthesize evidence on cultural, social, and psychological factors influencing access to diagnosis, treatment, and management of HIV, TB, and HIV-TB co-infection among immigrant and refugee populations in the United States. This study is an integrative review guided by Whittemore and Knafl’s framework. We conducted a systematic search of PubMed, Scopus, and CINAHL for peer-reviewed, mixed-methods, quantitative, qualitative, and review articles published in English between 2015 and 2025. After duplications were removed and dual screening of 107 retrieved records, 16 articles met inclusion criteria for this study. Findings were thematically categorized and synthesized. The themes generated were placed into three main categories: cultural factors including stigma and misperceptions about disease transmission; social factors such as limited healthcare access, lack of insurance, and socioeconomic marginalization; and psychological factors including fear of deportation, trauma, and mistrust of healthcare systems. Addressing HIV-TB co-infection in immigrant and refugee populations requires holistic, trauma-informed, and community-engaged strategies that integrate mental health support, culturally and linguistically tailored education, and policy reforms to remove structural obstacles. Future research should employ culturally validated measures to co-create sustainable interventions and advance health equity.

## 1. Introduction

Human Immunodeficiency Virus and Tuberculosis (HIV-TB) co-infection remains a critical public health challenge in the United States, particularly among immigrant and refugee populations. While effective antiretroviral therapy has transformed HIV into a manageable chronic disease, Mycobacterium tuberculosis (TB) is still the leading cause of death among people living with HIV, and co-infected individuals are at substantially higher risk of morbidity and mortality than those with either infection alone. In the United States, more than 70% of reported TB cases occur in non-U.S.-born populations and roughly 90% of these cases arise from reactivation of latent TB infection acquired abroad [1]. Concurrently, non-U.S.-born adults accounted for 16.2% of new HIV diagnosis during 2007–2010, despite comprising only 12.9% of the population [2,3]. This dual burden underscores the need to address HIV-TB co-infection in the context of social factors, which are conditions in which people are born, grow, live, work, and age, including socioeconomic status, social support networks, access to resources that influence outcomes [4], and psychological factors. These encompass individual-level mental and emotional processes such as trauma, stress, beliefs, attitudes, and self-efficacy that affect health behaviors and treatment adherence [5]. Within these communities, language limitations and fragmented healthcare access usually increase co-infection burdens and complicate prevention, diagnosis, and care.

Tuberculosis is caused by *Mycobacterium tuberculosis*, a bacterial pathogen transmitted through airborne droplets that primarily affect the lungs but can also involve other organs. The relationship between HIV and TB is biologically synergistic. HIV infection weakens the immune system, particularly by depleting Cluster of Differentiation 4-positive T lymphocytes (CD4+ T cells), thereby increasing the risk of progression from latent TB infection to active disease. Conversely, TB infection can accelerate HIV disease progression by increasing immune activation and viral replication. This bidirectional interaction contributes to higher morbidity and mortality among individuals with HIV-TB co-infection compared to those with either condition alone.

Immigrant and refugee populations in the United States bear a disproportionate share of HIV-TB co-infection, creating a perfect storm for HIV-TB co-infection in the U.S. According to the International Organization of Migration (IOM), an immigrant is a person who moves away from his or her place of usual residence, whether within a country or across an international border, temporarily or permanently, and for a variety of reasons [6]. In contrast, refugees are compelled to flee their country due to persecution, conflict, or war. Culture, the shared system of beliefs, values, norms, and practices that shape how groups understand health, illness, and care-seeking behaviors [7], deeply influences how these populations or groups experience migration. In 2020, there were 281 million immigrants in the world, of which approximately 3.6% of the world population lived outside their usual residences [8]. By 2024, the number of international migrants globally stood at 304 million, a figure that has doubled since 1990, when there were an estimated 154 million international immigrants globally. The U.S. hosted more international immigrants than any other country in 2024, with 52.4 million. Germany was the second leading destination for international immigrants, hosting 16.8 million in 2024, followed by Saudi Arabia (13.7 million), the United Kingdom (11.8 million), France (9.2 million), Spain (8.9 million), Canada (8.8 million), the United Arab Emirates (8.2 million), Australia (8.1 million), the Russian Federation (7.6 million), Türkiye (7.1 million), and Italy (6.6 million) [9]. In addition, culture influences how immigrant and refugee populations engage with health systems.

Cultural stigma, legal exclusions, and economic constraints critically shape care-seeking and retention in HIV-TB co-infection services. HIV-TB co-infection-related stigma prevents individuals from testing and undermines treatment adherence [10]. Immigrant-focused mixed-methods research identified three domains which include fear of deportation, lack of insurance, and cultural beliefs as key determinants of linkage to and retention in HIV care [11]. Qualitative studies with refugee cohorts further reported several domains such as language barriers, distrust of medical institutions, and trauma-related mental health challenges that delay both diagnosis and sustained treatment for HIV-TB co-infection [12]. By synthesizing quantitative and qualitative findings across the preceding domains, this integrative review will focus on identifying and describing different drivers of HIV-TB co-infection disparities and inform culturally consistent strategies to reduce morbidity and mortality in U.S. immigrant and refugee populations.

HIV and tuberculosis represent a uniquely synergistic co-infection of major public health concern. Tuberculosis is the leading cause of death among people living with HIV globally, and HIV infection significantly increases the risk of progression from latent to active TB. Co-infected individuals experience worse clinical outcomes, including higher morbidity and mortality, compared to those with either infection alone. In the United States, this burden is disproportionately concentrated among immigrant and refugee populations, where structural, cultural, and healthcare access barriers further complicate prevention, diagnosis, and management.

The purpose of this review was to analyze cultural, social, and psychological factors that influence access to and engagement with HIV and TB diagnosis, treatment, and management, with implications for HIV-TB co-infection among immigrant and refugee populations in the United States. Given the limited number of studies explicitly focused on HIV-TB co-infection in this population, a broader approach was adopted to capture shared determinants relevant to co-infection contexts. The overall goal is to understand the current state of the science regarding evidence-based insights that can help healthcare providers, policymakers, and researchers develop more effective, culturally appropriate interventions and policies and ultimately improve health outcomes for these communities.

## 2. Methods

Guided by Whittemore and Knafl’s integrative review approach (2005), which provides a comprehensive understanding of a particular healthcare phenomenon or problem to inform practice and policy, the following databases were searched on 10 and 11 June 2025: PubMed, Scopus, and Cumulative Index to Nursing and Allied Health Literature (CINAHL). This study adopts an integrative review approach, which allows for the inclusion and synthesis of diverse evidence sources, including quantitative, qualitative, and review studies, to provide a comprehensive understanding of complex healthcare phenomena. Table 1 presents the full list of keywords and search strings. Search terms were made broad and inclusive to account for variations in electronic databases. We also manually searched the reference lists of the retrieved articles for additional citations. We excluded from the search gray literature, non-English studies, studies that focused on animals, or studies published outside the United States. Studies with mixed populations (i.e., immigrants and asylum seekers) were included in the search. The search strategy was intentionally designed to be broad by including studies on HIV and/or tuberculosis to capture shared cultural, social, and psychological determinants relevant to HIV-TB co-infection. Given the limited number of studies explicitly examining co-infection in this population, this approach allowed for a more comprehensive synthesis of factors influencing care.

While the review focused on HIV-TB co-infection, relatively few studies explicitly examined co-infection outcomes. Therefore, studies addressing HIV or TB independently were included when they provided relevant insights into shared structural, cultural, and psychological determinants that influence co-infection risk and care.

### 2.1. Study Selection and Reporting

**Figure 1:**
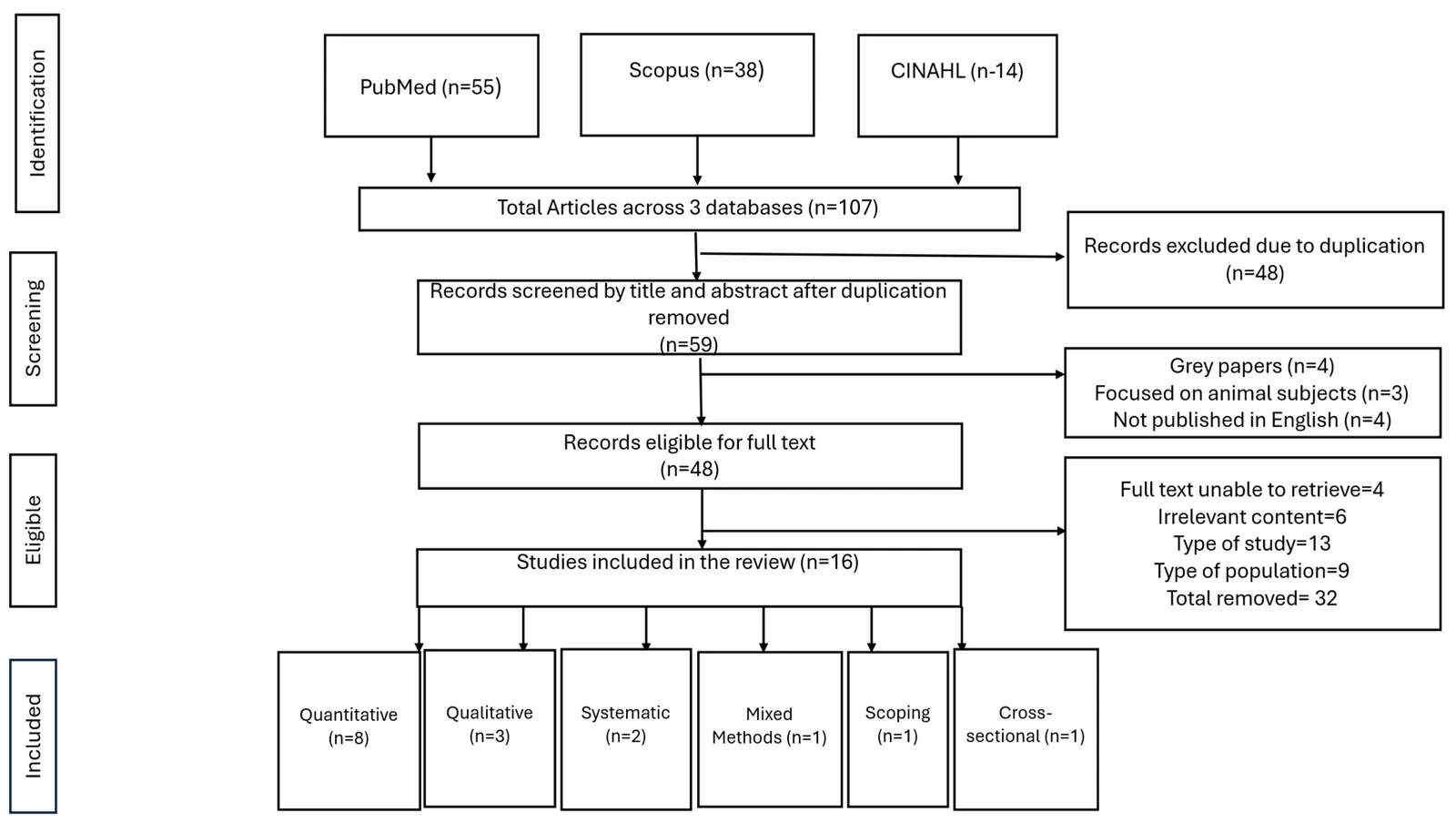
Flowchart of the literature review process.

The study selection and reporting process followed the Preferred Reporting Items for Systematic Reviews and Meta-Analyses (PRISMA) guidelines. Titles and abstracts were independently screened by two reviewers, followed by full-text assessment to determine eligibility. Discrepancies were resolved through discussion. The study selection process is summarized in a PRISMA flow diagram (Figure 1). This review adheres to PRISMA reporting standards to enhance transparency and reproducibility. This review was conducted and reported according to the PRISMA 2020 guidelines; the completed PRISMA checklist is provided in Table S1 in the Supplementary Material.

To minimize potential duplication of findings arising from the inclusion of review articles, we carefully examined primary studies referenced within included reviews and avoided double-counting evidence during synthesis. Findings were interpreted at a conceptual level rather than aggregated quantitatively, reducing the risk of bias due to overlap.

### 2.2. Data Extraction and Analysis

Figure 1 describes the data extraction and analysis process. Citations from each literature search were imported into EndNote reference manager software. After duplicate citations were removed, two public health specialists (PA and KK) independently screened the titles and abstracts of the articles to determine their relevance and whether they met the inclusion criteria. Disagreements were discussed to reach a consensus. The full texts of relevant articles, or articles for which relevance could not be ascertained by the title and abstract screening, were retrieved and reviewed to further determine eligibility. For each study included, PA and KK independently extracted data about the study design, aim of study, sample description, impact of diagnosis, care management, and study findings. Study finding data were categorized as prevalence and patterns of cultural, social, and psychological factors that prevent immigrants and asylum seekers in the U.S. from accessing diagnosis and treatment of HIV-TB co-infection. The data extracted by PA and KK were integrated into a single data matrix. Following the methodology developed by the data matrix was iteratively compared to each category of findings across studies to summarize and synthesize the data. Synthesized data were then reviewed to make sure they matched and accurately reflected what the original studies reported.

### 2.3. Inclusion Criteria

Research articles related to HIV-TB co-infections among immigrants and refugee populations in the U.S. were included in this review. First, the titles of the articles were examined, and duplicate articles were excluded from the study. Among the remaining articles, the abstracts of the articles that met the inclusion criteria below were independently reviewed by two members of the research team and included in our review. Studies were included if they examined HIV, tuberculosis, or both, provided they reported cultural, social, or psychological factors influencing access to, engagement with, or management of care. Studies focusing exclusively on unrelated conditions were excluded. We included studies that

- Employed a mixed-methods design that incorporates both qualitative and quantitative data, and reviews.
- Included HIV and tuberculosis co-infection among immigrant and refugee populations in the United States.
- Were published between 2015 and 2025.
- Were peer-reviewed in English.
- Focused on human subjects.
- Were published in the United States.

Although the search strategy was broad, studies were included only if they provided empirical evidence on cultural, social, or psychological factors directly influencing access to, engagement with, or outcomes of HIV or tuberculosis-related care. Studies that only mentioned these conditions without examining determinants of care, behavior, or health system interaction were excluded. For example, studies examining healthcare access, insurance status, or health beliefs among immigrant populations were included when they provided transferable insights into structural and behavioral factors relevant to HIV and TB care.

Review articles were included only if they provided synthesized insights directly relevant to cultural, social, or psychological determinants of HIV or tuberculosis care and did not duplicate findings already captured from primary studies included in this review.

### 2.4. Quality Assessment

The Critical Appraisal Skills Program (CASP) checklist was used to check the quality of the studies (Table 2). Based on the CASP checklist, all the studies were of suitable quality to be included in the integrative review if the ranking was 5–7 (Moderate) or 8–10 (High). This review was not registered in a systematic registry including PROSPERO, and a protocol was not published prior to the conduct of the study.

## 3. Results

A total of 16 studies met the inclusion criteria (Figure 1). Across these studies, three major domains were identified: cultural, social, and psychological factors influencing HIV and tuberculosis-related care among immigrant and refugee populations. Stigma emerged as a cross-cutting factor across all domains, shaping healthcare access, diagnosis, and treatment engagement.

Three major domains were identified, cultural, social, and psychological factors, with stigma emerging as a cross-cutting influence across all domains. Across studies, findings showed both consistency and variation in how cultural, social, and psychological factors influenced HIV and TB care, with differences observed across populations and study contexts.

The searches from the three (3) electronic databases from 10 June to 14 June 2025, produced a total of 107 articles (PubMed = 55, Scopus = 38, and CINAHL = 14). Figure 1 depicts the search, selection, and exclusion process using a PRISMA flow diagram. Sixteen articles were included in the analysis (Table 2); the majority of articles were quantitative studies. The characteristics and key findings of the included studies are summarized in Table S2 in the Supplementary Material. The themes generated from the included articles were categorized into cultural, social, and psychological factors. While cultural, social, and psychological factors are presented as distinct domains for analytical clarity, several key influences particularly stigma, fear, and mistrust emerged as cross-cutting factors operating across multiple levels. Cultural factors primarily reflect shared beliefs, norms, and meanings related to illness; social factors capture structural and systemic conditions such as access to care and socioeconomic status; and psychological factors represent individual-level perceptions and emotional responses. Stigma emerged as a cross-cutting factor influencing cultural, social, and psychological domains. A quality assessment of all 16 articles was conducted.

The included studies represented diverse immigrant populations and were categorized into five main groups: Latino/Hispanic immigrant populations (n = 2), Asian immigrant populations (n = 1), mixed immigrant populations (non-U.S.-born individuals) (n = 4), refugees and asylum seekers (n = 2), and specialized high-risk subpopulations such as migrant workers, sex workers, and children (n = 5). An additional subset of studies (n = 2) focused on general immigrant or refugee populations within intervention or health system contexts (Table 3). The majority of studies focused on Latino and mixed immigrant populations, while relatively few studies examined Asian populations or other immigrant subgroups, highlighting potential gaps in the literature. Categories are mutually exclusive and each study was assigned to the most representative population group based on the primary study focus.

### 3.1. Cultural Factors

Cultural beliefs and norms significantly influence health-seeking behavior, diagnosis, and treatment adherence among immigrants and refugees affected by HIV-TB co-infection in the United States. The results indicated multiple inter-related cultural barriers that impact effective diagnosis, treatment, and management of these comorbidities, which range from misperceptions of HIV and TB, deeply entrenched stigma, and systemic distrust, which shapes every stage of the healthcare continuum from diagnosis to long-term management [18,22].

Regarding the misconceptions about TB and HIV transmission among non-U.S.-born individuals, many non-U.S.-born individuals, particularly those from TB-endemic countries, perceived TB as a hereditary illness or one spread through casual contact like sharing utensils, rather than through airborne particles [2,23]. Similarly, misunderstandings about the protective value of the Bacillus Calmette–Guérin (BCG) vaccine, often received in their home countries, led some immigrants to distrust TB diagnostic tests in the U.S., particularly the tuberculin skin test (TST), which is believed to yield false positive results due to prior vaccination [2,23]. These misperceptions resulted in delayed or missed diagnoses, diminished trust in the U.S. healthcare system, and reluctance to initiate or complete treatment for latent TB infection.

Anticipated and experienced stigma is another recurring cultural factor identified that negatively affects care. In many immigrant communities, TB-HIV co-infections are perceived as markers of moral failure, sexual promiscuity, or divine punishment leading to social isolation and secrecy [17,21]. Among women and sex workers, the stigma is often compounded by gendered cultural norms and occupational taboos, making disclosure and treatment adherence difficult [17]. This fear of ostracism frequently outweighs the perceived benefit of engaging in early care and contributes to disengagement from formal healthcare services [18].

Cultural norms that devalue preventive healthcare further impact effective diagnosis, treatment, and management. In several communities, particularly among Latino immigrants, health interventions are sought only when symptoms become severe, while latent or asymptomatic infections are often overlooked [15,20]. This belief system reduces the perceived relevance of early diagnosis or preventive treatment of HIV-TB co-infection, finding that non-linkage to HIV care was particularly common when individuals were tested in impersonal settings like emergency rooms or when health messages lacked cultural relevance [15].

While cultural factors such as stigma and health beliefs were consistently reported across studies, their manifestation varied by population. For example, stigma in Latino populations was often linked to community perceptions and social identity, whereas in refugee populations it was more closely tied to trauma and displacement experiences.

**Mistrust of the healthcare system** has been reported across studies and shapes every stage, rooted in experiences of discrimination or exclusion in both the U.S. and countries of origin, further increasing these challenges [18,22]. Refugees and asylum seekers frequently perceived targeted health messaging, such as highlighting TB prevalence in their country of origin, as xenophobic or punitive [2,18,22]. Overall, this sense of being “targeted” discourages participation in voluntary screening programs and diminishes engagement with HIV-TB co-infection services.

Moreover, **language barriers and inadequate health communication** significantly contribute to health disparities. Immigrants with limited English proficiency often struggle to comprehend medical instructions, understand risk messaging, and complete the bureaucratic steps required for care [2,20]. Without cultural and linguistically adapted education and navigation support, misinformation and non-adherence become more likely [24]. In many cultural groups that prioritize the needs, goals, and well-being of the group, family and community reputation heavily influence individual health decisions. The fear of bringing shame or dishonor to the family can prevent individuals from seeking timely diagnosis or disclosing their condition [13,21,25]. In such settings, community opinions and traditional healing methods may take precedence over biomedical treatment, further complicating adherence and continuity of care.

Finally, **cultural thresholds for illness perception** affect care-seeking behavior. In many cultures, illness is not acknowledged until it visibly disrupts daily functioning. Consequently, early phases of HIV and latent TB infection are not considered serious enough to warrant medical attention, delaying initiation of therapy and contributing to co-infection complications [2,23].

Together, these interwoven cultural factors create a web of barriers that prevent timely and equitable care for HIV-TB co-infection.

### 3.2. Social Factors

Several studies described a complex web of socioeconomic, institutional, and structural challenges that inhibit timely diagnosis, disrupt treatment adherence, and undermine effective management of HIV-TB co-infections. Access to healthcare remains a persistent and multidimensional barrier. Many immigrants and refugees, particularly those who are undocumented or recently migrated, lack health insurance or a regular source of care. Moreover, those with insurance encounter systemic barriers navigating a fragmented, often culturally mismatched healthcare system [1]. Despite having some form of coverage, non-U.S.-born individuals report significant challenges in maintaining continuity of care for HIV-TB co-infections, and related services [18,22]. These disparities are exacerbated by cultural factors such as limited availability of linguistically appropriate and culturally sensitive care models, which can isolate patients and discourage them from seeking or remaining in healthcare [19].

Immigration status and the broader sociopolitical context surrounding immigration enforcement contribute significantly to healthcare avoidance. Fear of deportation, especially among undocumented individuals, often results in the under-utilization of public health services, even when these services are offered at no cost [18,22]. Immigrants may interpret TB screening campaigns that highlight the prevalence of TB in their countries of origin as xenophobic or stigmatizing, thereby discouraging participation [2]. Similar perceptions were noted among asylum seekers, who described feeling targeted and dehumanized by health assessments that emphasized their nationality [18,22]. These experiences deepen mistrust toward the healthcare system and delay engagement with services that are critical for early diagnosis and treatment.

**Socioeconomic marginalization** including poverty, housing instability, food insecurity, and informal employment further compounds access issues. Many immigrants prioritize daily survival over preventive health, leading to delays in care-seeking and poor adherence to treatment. For instance, reference [15] observed that Latino individuals living in lower-income or less ethnically concentrated neighborhoods were significantly less likely to link to HIV care following a diagnosis. Additionally, immigrant farm workers, who often work long hours and live in crowded, unsanitary conditions, encounter logistical obstacles such as transportation difficulties and inflexible work schedules. Reference [23] emphasized that traditional healthcare models often fail to accommodate such populations, and that mobile clinics and community-based testing are essential for reaching them.

**Institutional and structural discrimination** also influences the trajectory of HIV-TB co-infection care. Reference [16] reported that while foreign-born individuals living with HIV had relatively better antiretroviral therapy prescription and viral suppression outcomes compared to their U.S.-born counterparts, their care experiences were shaped by broader systemic inequities, including racism and healthcare bias. These experiences may be re-traumatizing for individuals who have fled persecution or conflict, and they contribute to social alienation that weakens engagement with healthcare providers.

**Health communication** remains formidable barriers to care. Limited English proficiency hinders patients’ ability to navigate complex health systems, understand medical instructions, and access culturally relevant information. Public health campaigns that fail to incorporate multilingual, community-informed approaches often fall short of their intended impact. Reference [19] found that the Centers for Disease Control’s (CDC) “Think. Test. Treat TB” campaign did not adequately reach immigrant populations, due to insufficient cultural tailoring, reducing its effectiveness in promoting TB awareness and testing. Social isolation and the absence of support networks further complicate healthcare access and engagement. Many migrants and refugees live without family or community support, which may otherwise help them navigate medical systems or reinforce treatment adherence. In tightly knit communities, stigma surrounding HIV and TB is particularly damaging, as individuals may conceal their status to avoid moral judgment or ostracism [20,23]. This fear frequently outweighs the perceived benefits of early intervention and leads to significant delays in diagnosis and treatment. Conversely, studies have demonstrated the potential of the community-based participatory research (CBPR) approach in improving health literacy and reducing stigma. Reference [24] found that CBPR-informed interventions enhanced TB screening uptake among Latino immigrants by building trust and aligning outreach with community values. These findings underscore the profound impact of social context on the HIV-TB co-infection continuum of care among immigrants and refugees.

Although structural barriers such as lack of insurance and socioeconomic disadvantage were widely reported, some studies identified relatively high levels of healthcare engagement among non-U.S.-born populations, suggesting that access may be influenced by program availability and community support.

### 3.3. Psychological Factors

Psychological factors play a foundational role in shaping the health-seeking behaviors and treatment trajectories of migrants and refugees co-infected with HIV and TB in the U.S. These factors ranging from trauma and chronic stress to internalized stigma and low self-efficacy intersect with structural barriers to deepen vulnerability among non-U.S.-born populations. They influence every stage of the healthcare continuum, from diagnosis through long-term management.

A consistent finding is the psychological burden stemming from trauma, displacement, and the process of migration. Migrants and refugees often arrive in the U.S. having survived war, persecution, forced migration, and family separation that heighten susceptibility to mental health disorders such as anxiety, depression, and post-traumatic stress disorder (PTSD). Reference [23] reported that migrant farmworkers on the U.S.–Mexico border experienced high levels of psychological distress, which, compounded by social and physical isolation, undermined motivation to pursue screening or treatment for latent TB infection or HIV. The cumulative emotional strain of adjusting to new environments, language, and cultural systems further exacerbates disengagement from care.

Fear, particularly of deportation, criminalization, and public exposure, acts as a profound deterrent to accessing HIV and TB services. Among undocumented migrants and asylum seekers, healthcare institutions are often perceived not as safe havens but as potential threats. Studies reported that this fear is not only rooted in immigration status but also shaped by public health narratives that stigmatize migrants as disease carriers. For instance, reference [2] found that public health campaigns highlighting the high TB-HIV co-infection burden in migrants’ countries of origin were interpreted as xenophobic by some participants, reinforcing psychological resistance to screening and treatment programs. Similarly, reference [18] described how asylum seekers expressed feelings of re-traumatization and alienation during TB screening procedures, experiences that triggered past trauma and eroded trust in medical institutions.

Internalized stigma and anticipated social judgment further compound psychological distress. HIV and TB remain heavily stigmatized in many migrant communities, where they are often viewed through moral, religious, or fatalistic lenses. Individuals may interpret a diagnosis as evidence of personal failure or divine punishment, leading to shame, secrecy, and social withdrawal. Reference [15] reported that Latinos living in areas with low ethnic density were particularly prone to non-linkage to HIV care due to fears of community judgment and cultural isolation. Similarly, reference [25] documented that adolescents and caregivers living with HIV expressed intense shame around TB preventive therapy, resulting in poor adherence to isoniazid due to low confidence, confusion about dosing, and fear of side effects.

Acculturative stress and cultural dissonance are also significant psychological burdens for immigrants. Navigating unfamiliar health systems, reconciling different health beliefs, and coping with cultural alienation create chronic emotional fatigue [7]. Migrants who feel misunderstood or unsupported by healthcare providers may internalize feelings of helplessness, further reducing their willingness to pursue care. Learned helplessness, a condition in which individuals perceive that their efforts have no positive impact [16], may develop after repeated failures to access culturally competent care, resulting in disengagement from both preventive and curative services.

Additionally, the absence of extended family, religious networks, or culturally grounded community resources magnifies psychological isolation. This isolation weakens coping mechanisms and removes essential buffers against mental health decline. Without trusted support, many migrants internalize blame and avoid healthcare spaces altogether [13,20,25].

## 4. Discussion

This integrative review examined the cultural, social, and psychological determinants influencing the diagnosis, treatment, and management of HIV-TB co-infection among immigrant and refugee populations in the United States. The synthesized findings corroborate established literature while also highlighting important areas of divergence and previously underexplored gaps. Additionally, the review demonstrates that cultural, social, and psychological factors are key determinants of HIV and TB care among immigrant populations; however, their influence is not uniform. Instead, these factors interact differently across populations and contexts, highlighting the need for context-specific approaches to care.

The findings across included studies demonstrate both consistency and variation in the factors influencing HIV-TB co-infection care. While cultural stigma and psychological barriers were consistently reported across studies, the magnitude and manifestation of these factors varied depending on context, population characteristics, and healthcare systems. Some studies emphasized delays in diagnosis, whereas others highlighted challenges in treatment adherence and continuity of care. Additionally, variations in study design and setting contribute to differences in reported outcomes. The overall evidence base is largely composed of qualitative and cross-sectional studies, which provides valuable contextual insight but may limit generalizability and causal interpretation. These differences underscore the importance of context-specific and multi-level approaches in addressing HIV-TB co-infection. These findings highlight that barriers to HIV-TB care among immigrant and refugee populations are not isolated but operate across multiple levels, with stigma emerging as a cross-cutting factor that influences healthcare engagement, disclosure, and treatment adherence.

The distribution of the included studies likely reflects the geographic concentration of research in Latino-dense regions of the United States rather than a limitation of the search strategy. However, it highlights an important gap in the literature, with fewer studies examining other immigrant populations such as African, Asian, and Middle Eastern communities.

Consistent with prior studies, this review demonstrates that stigma both perceived and enacted remains a central barrier to engagement across the HIV-TB care continuum [17,21]. Cultural misconceptions surrounding HIV and TB, particularly those that frame these conditions as moral or social failings, were frequently cited as reasons for delayed testing, low treatment adherence, and disengagement from formal care systems. Among women and sex workers, stigma was often compounded by gendered cultural norms, resulting in heightened barriers to disclosure and sustained treatment [17]. These findings align with broader evidence suggesting that intersectional stigma where multiple stigmatized identities overlap can significantly reduce healthcare engagement.

These findings also highlight the central role of structural barriers in shaping access to HIV and TB care, suggesting that interventions targeting individual behavior alone may be insufficient without addressing systemic inequities. These findings are supported by [1], who identified a high prevalence of latent TB infection among non-U.S.-born individuals despite substantial gaps in access to usual sources of care. Psychological determinants, particularly trauma histories, fear of deportation, and low self-efficacy, further compounded barriers to care. These were echoed across multiple studies in this review, including those by [18,22], underscoring the need for trauma-informed and psychologically responsive care models.

The results also align with [3], who identified widespread misperceptions about TB transmission, the role of the Bacillus Calmette–Guérin (BCG) vaccine, and the purpose of LTBI testing. Many participants believed TB could be transmitted via casual contact or shared utensils, and some assumed prior BCG vaccination conferred lifelong immunity. Such misbeliefs were associated with distrust in TB diagnostic protocols and hesitancy to initiate or adhere to treatment regimens. These findings further illustrate the critical need for culturally tailored education to address knowledge gaps and reduce stigma.

While stigma emerged as a cross-cutting factor across studies, its expression differed by population. For example, studies among Latino immigrants emphasized stigma related to community norms and social identity, whereas studies among refugee populations highlighted stigma shaped by trauma and displacement. Similarly, structural barriers such as access to care were widely reported, but some studies identified relatively high engagement in care among non-U.S.-born populations, suggesting that healthcare access may be mediated by programmatic support and local healthcare infrastructure.

Despite the alignment with existing literature, this review also identified several underexplored areas. Notably, the influence of intersectional stigma remains inadequately measured in current empirical studies, despite strong qualitative evidence of its impact. Additionally, while many studies highlighted the role of family and community norms, few examined the implications of collectivist cultural values on decision-making regarding HIV-TB care. This gap limits the development of culturally congruent interventions that account for social and familial dynamics in health-seeking behavior. The reliance on broader HIV and TB literature reflects a critical gap in co-infection-specific research in U.S. immigrant populations.

Contradictions were also evident across studies. For example, reference [16] reported that non-U.S.-born individuals had higher rates of ART adherence and viral suppression compared to U.S.-born individuals, potentially due to greater access to Ryan White services. This finding appears at odds with qualitative evidence suggesting systemic barriers reduce care continuity for immigrants. These discrepancies point to the need for disaggregated data that account for variables such as legal status, time since immigration, and access to culturally specific care programs.

Findings regarding healthcare access and engagement were not entirely consistent across studies. While several studies reported significant barriers due to lack of insurance and socioeconomic constraints, others observed relatively high levels of care engagement among certain immigrant groups. These differences suggest that access to care may be context-dependent and influenced by local resources, policy environments, and community support systems.

Similarly, while targeted messaging has been shown to increase TB screening intentions [19,24], findings from [3] suggest that such campaigns may be perceived as discriminatory or stigmatizing if not framed with cultural sensitivity. These mixed findings indicate the importance of co-developing communication strategies with community stakeholders to ensure they are both effective and respectful.

By synthesizing evidence across diverse study designs and populations, this review provides a multi-level understanding of how cultural, social, and psychological factors interact to shape HIV and TB care. Unlike individual studies, this integrative approach highlights the interconnected nature of these determinants and their implications for co-infection contexts. A key contribution of this review is its integrative approach, which synthesizes evidence across diverse study designs and populations to provide a multi-level understanding of determinants of HIV and TB care. Unlike individual studies, this review highlights how cultural, social, and psychological factors interact to shape care engagement, offering a more comprehensive framework for understanding co-infection-related challenges. Most included studies were of moderate to high quality based on CASP appraisal; however, the predominance of cross-sectional designs limits the causal interpretation of findings. Additionally, several studies relied on self-reported data, which may introduce recall or social desirability bias. These factors should be considered when interpreting the strength of the evidence.

Although this review focused on HIV-TB co-infection, relatively few studies explicitly examined co-infection among immigrant and refugee populations in the United States. As a result, we included studies addressing HIV or tuberculosis independently when they provided relevant insights into shared determinants. While this approach broadens understanding, it may limit the specificity of conclusions directly attributable to co-infection.

Another limitation to this study is that while the search strategy yielded a broad pool of studies, inclusion was restricted to those that provided conceptually relevant insights into determinants of care. This selective approach may have excluded studies with more distal or indirect relevance, but it was necessary to maintain analytical focus.

The inclusion of both primary and review studies may introduce potential overlap in reported findings. However, this was mitigated by synthesizing evidence at a conceptual level and avoiding duplication of results during analysis.

Also, the findings from the included studies may be more reflective of populations that are more frequently studied, potentially limiting the generalizability of conclusions to underrepresented immigrant groups.

### Recommendations

The findings of this integrative review highlight the need for comprehensive, culturally responsive, and trauma-informed approaches to HIV-TB co-infection care among immigrant and refugee populations in the United States. Health systems must move beyond biomedical interventions and adopt integrated models of care that account for the intersecting cultural, social, and psychological challenges faced by these communities. Care delivery should be linguistically accessible, culturally sensitive, and explicitly traumatically informed, reflecting the complex lived experiences of individuals who often navigate both chronic disease and migration-related adversities. Studies have demonstrated that culturally grounded programs, particularly those incorporating mental health support and language-concordant services, can improve both engagement and adherence to treatment [18,22].

Equally critical is the co-creation of health communication strategies with affected communities. This literature underscores the risks of stigmatization when messaging is perceived as targeting or blaming certain nationalities or cultural groups [3]. Public health communication efforts should therefore be collaboratively designed to ensure that messages are not only medically accurate but also culturally affirming and resonant. Clarifying the implications of latent TB infection, the limitations of the Bacillus Calmette–Guérin (BCG) vaccine, and the need for testing even among asymptomatic individuals can dispel prevalent misconceptions and support more informed decision-making.

In addition, expanding access to community-based navigation services can mitigate many of the logistical and bureaucratic barriers to care. Evidence from participatory interventions suggests that bilingual and culturally matched community health workers are particularly effective in building trust and increasing care engagement among marginalized immigrant groups [24]. Their roles as cultural brokers, health educators, and system navigators are essential in addressing structural barriers such as insurance enrollment, transportation, and clinic accessibility.

From a policy perspective, structural reforms are essential to dismantling systemic barriers that perpetuate disparities in HIV-TB outcomes. Policies that ensure equitable healthcare access, irrespective of immigration status, are foundational to reducing the burden of co-infection. Safeguarding patient data from immigration enforcement and investing in wraparound services—such as housing, food assistance, and mental health support—are critical to sustaining treatment adherence and improving long-term outcomes [1,16]. These structural determinants must be prioritized alongside clinical care to effectively address the root causes of disparities.

Finally, ongoing research should prioritize longitudinal, disaggregated, and community-engaged methodologies. Many of the studies reviewed highlight the need for more nuanced data that captures heterogeneity within immigrant and refugee populations. Disaggregating by country of origin, immigration pathway, length of U.S. residency, and legal status can illuminate context-specific barriers and inform targeted interventions. Longitudinal research is especially necessary to evaluate the sustainability of interventions and track outcomes over time. Moreover, involving communities as partners in the research process can ensure that study designs and interventions are both ethical and relevant to those most affected.

Together, these recommendations emphasize the importance of addressing HIV-TB co-infection not only as a clinical challenge but also as a reflection of broader social and structural inequities. A shift toward equity-centered, community-engaged, and structurally informed approaches is essential for advancing health outcomes among immigrant and refugee populations in the U.S.

## 5. Conclusions

This integrative review highlights that HIV-TB co-infection among immigrant and refugee populations in the United States is shaped by interconnected cultural, social, and psychological factors that influence access to diagnosis, treatment, and continuity of care. While biomedical advances have improved clinical outcomes, their impact remains limited when structural and sociocultural barriers such as stigma, language discordance, limited health literacy, and immigration-related vulnerabilities are not addressed.

Addressing these disparities requires a clear, multi-level intervention framework. At the community level, culturally and linguistically tailored health education and peer-based navigation programs can improve trust, engagement, and care uptake. At the health system level, integrating HIV-TB services with mental health and social support, alongside culturally competent care, can enhance continuity and treatment adherence. At the policy level, addressing structural determinants such as healthcare access, immigration-related fears, and socioeconomic instability is essential to reduce inequities.

This review contributes to the field by emphasizing the need for integrated, context-sensitive approaches that move beyond isolated interventions and instead address the intersecting realities shaping health behaviors and outcomes. Future research should prioritize community-engaged and co-designed interventions that reflect the lived experiences of immigrant and refugee populations.

## Figures and Tables

**Table 1:** Database search strings.

Database	Search String	Total Articles Retrieved
PUBMED		55
	(“HIV”[Mesh] OR “Human Immuno Deficiency Virus*” AND “Tuberculosis”[Mesh] OR tuberculosis OR tb) AND (“Refugees”[Mesh] OR “Undocumented Immigrants”[Mesh] OR migrant* OR “undocumented migrant*” OR “asylum seeker*” OR immigrant*) AND (“Social Determinants of Health”[Mesh] OR (MM “Psychology+”) OR “Social Factors”[Mesh] OR “Healthcare Disparities”[Mesh] OR attitude* OR belief* OR thought* OR behavior* OR behaviour* OR “healthcare access”) AND (“United States”[Mesh] OR “united states” OR “the united states” OR “the united states of America” OR “the u.s.”)	
Scopus		38
	TITLE-ABS-KEY(HIV OR “human immunodeficiency virus”) AND TITLE-ABS-KEY(TB OR tuberculosis) AND TITLE-ABS-KEY(psychological OR psychosocial OR social OR “social determinants” OR “social factor*” OR “healthcare disparit*”) AND TITLE-ABS-KEY(immigrant* OR refugee* OR “undocumented immigrant*” OR migrant*) AND TITLE-ABS-KEY(“united states” OR US OR USA OR America) AND (LIMIT-TO (DOCTYPE,”ar”)) AND (LIMIT-TO (LANGUAGE,”English”))	
CINAHL		14
	((MM “Human Immunodeficiency Virus+”) OR HIV OR “human immunodeficiency virus”) AND ((MM “Tuberculosis+”) OR tb OR tuberculosis) AND ((MM “Undocumented Immigrants”) OR “Immigrant health” OR (MM “Refugees+”) OR immigrant* OR refugee* OR “undocumented immigrant*” OR migrant*) AND ((MM “Social Determinants of Health”) OR (“MM Culture+”) OR (MM “Psychology+”) OR (MM “Social Support+”) OR (MM “Social Factors”) OR (MM “Health Status Disparities+”) OR psychological OR psychosocial OR social OR “social determinants” OR “social factor*” OR “healthcare disparit*”) AND ((MM “United States+”) OR “united states” OR US OR USA OR America)	
Total		107
Duplicates Removed		48
Total after Duplication		59

**Table 2:** Critical Appraisal Skills Program checklist table.

No	Author	Year	Study Design	Appraisal Summary	Quality Appraisal
1	[13]	2020	Cohort	Yes (6), No (1), Can’t tell (3)	Moderate
2	[1]	2021	Cohort	Yes (9), No (1), Can’t tell (1)	High
3	[14]	2021	Cohort	Yes (9), No (1), Can’t tell (2)	High
4	[15]	2017	Cohort	Yes (8), No (1), Can’t tell (1)	High
5	[16]	2015	Cohort	Yes (7), No (1), Can’t tell (2)	Moderate
6	[12]	2024	Cohort	Yes (8), No (1), Can’t tell (1)	High
7	[17]	2018	Cohort	Yes (8), No (1), Can’t tell (0)	High
8	[18]	2018	Qualitative	Yes (8), No (2), Can’t tell (1)	High
9	[3]	2023	Qualitative	Yes (8), No (2), Can’t tell (1)	High
10	[2]	2022	Qualitative + Quantitative	Yes (8), No (2), Can’t tell (1)	High
11	[19]	2025	Mixed Methods	Yes (7), No (1), Can’t tell (3)	Moderate
12	[20]	2025	Mixed Methods	Yes (8), No (1), Can’t tell (2)	High
13	[21]	2016	Systematic Reviews	Yes (6), No (1), Can’t tell (1)	Moderate
14	[22]	2025	Systematic Reviews	Yes (9), No (2), Can’t tell (0)	Moderate
15	[10]	2025	Scoping Reviews	Yes (9), No (2), Can’t tell (0)	Moderate
16	[23]	2016	Cross-Sectional	Yes (9), No (2), Can’t tell (0)	Moderate

**Table 3:** Distribution of included studies by population group.

Population Group	Number of Studies (n)	Percentage (%)
Latino/Hispanic immigrant populations	2	12.5
Asian immigrant populations	1	6.25
Mixed immigrant populations (multiethnic/non-U.S.-born)	4	25.0
Refugees and asylum seekers	2	12.5
Specialized high-risk subpopulations (e.g., migrant workers, sex workers, children)	5	31.25
General immigrant/refugee populations (intervention or health system studies)	2	12.5
Total	16	100

## Data Availability

No new data were generated in this study. All data supporting the findings of this study are derived from previously published articles, which are cited within the manuscript.

## Supplementary Materials

The following supporting information can be downloaded at https://www.mdpi.com/article/10.3390/ijerph23080987/s1, Table S1: PRISMA checklist. Table S2: Summary of included articles [1,2,3,4,7,8,9,13,16,17,18,19,22,23,25,26].

## List of Abbreviations

HIV	Human Immunodeficiency Virus
TB	Tuberculosis
HIV-TB	Human Immunodeficiency Virus–Tuberculosis co-infection
ART	Antiretroviral Therapy
PRISMA	Preferred Reporting Items for Systematic Reviews and Meta-Analyses
CINAHL	Cumulative Index to Nursing and Allied Health Literature
CDC	Centers for Disease Control and Prevention
CD4	Cluster of Differentiation 4
IGRA	Interferon-Gamma Release Assay
TST	Tuberculin Skin Test
CBPR	Community Based Participatory Research
BCG	Bacillus Calmette–Guérin
LTBI	Latent Tuberculosis Infection
PTSD	Post-Traumatic Stress Disorder
