# Supplementary material for: HIV–Tuberculosis Co-Infection Among Immigrants and Refugees in the United States: An Integrative Review
Source: Int J Environ Res Public Health. 2026 Jul 29;23(8):987. doi: 10.3390/ijerph23080987 (PMC13513483; doi:10.3390/ijerph23080987)
Supplement: Supplementary file 1 [file ijerph-23-00987-s001.zip › ijerph-4222585-Table S2.pdf]

Table S2. Characteristics and Findings of the Included Studies.

Author and Year	Title	Variables	Study Design	Purpose	Limitations	Strengths	Key Findings	Population
Kay et al., 2020 [25]	Predictors of suboptimal adherence to isoniazid preventive therapy among adolescents and children living with HIV.	Cultural stigma, social barriers like cost and community judgment, and psychological challenges such as low self-efficacy and trauma collectively hinder the diagnosis, treatment, and management of HIV–TB co-infection among immigrant and refugee populations.	Quantitative study	Identify sociodemographic, psychosocial, and structural predictors of suboptimal IPT adherence to guide targeted interventions.	Small sample size with missing data, liberal Type I error rate, and single-site context limiting generalizability.	Validated, stakeholder-adapted survey in SiSwati, objective pill-count adherence measures, and high-performing risk score models.	22.5% of adolescents and 37.5% of children exhibited suboptimal adherence, driven by stigma, low self-efficacy, transport costs, and caregiver discomfort, with AUCs of 0.87 and 0.76.	Adolescents and children living with HIV

**Annan et al., 2021 [1]**	A cross-sectional study of latent tuberculosis infection, insurance coverage, and usual sources of health care among non-US-born persons in the United States.	Cultural unfamiliarity with the healthcare system, social barriers like lack of insurance and poverty, and psychological factors such as fear, stigma, and immigration-related stress hinder the diagnosis, treatment, and management of HIV–TB co-infection among immigrant and refugee populations in the United States.	Quantitative study	To assess health insurance coverage and usual sources of care among non-US-born people with latent tuberculosis infection in order to identify opportunities for targeted TB prevention within the private healthcare sector.	Data predates the Affordable Care Act implementation, excludes institutionalized and homeless populations, and lacks detailed country-of-origin information, potentially underestimating current insurance coverage and LTBI prevalence in subgroups.	Utilization of a nationally representative, complex-survey dataset with objective IGRA measurements and robust weighting methods enhances the validity and generalizability of findings.	An estimated 15.9% of non-US-born persons were IGRA-positive, of whom 63.0% had insurance and 74.1% had a usual source of care, especially high LTBI prevalence among Medicare beneficiaries (29.1%), indicating that private healthcare settings could reach most LTBI-positive individuals.	Non-US-born adults with latent TB infection

Author and Year	Title	Variables	Study Design	Purpose	Limitations	Strengths	Key Findings	Population
**Demeke et al., 2021 [16]**	Antiretroviral prescription, retention in care and viral suppression by place of birth among adults with diagnosed HIV in the United States-2015-2017, medical monitoring project.	Cultural stigma, social inequities, and psychological distress collectively reduce access to care, delay diagnosis, and hinder treatment adherence for HIV–TB co-infection among immigrant and refugee populations in the United States.	Quantitative study	To compare antiretroviral therapy prescription, retention in care, and viral suppression outcomes between US-born and non-US-born adults with diagnosed HIV in the United States.	The cross-sectional design prevents causal inference, self-reported measures may introduce bias, and data abstracted only from the usual care facility may not capture all services received.	Utilization of standardized medical record abstraction and multilingual interviews within a nationally representative complex- survey framework enhances the validity and generalizability of the findings.	Non-US-born adults had higher ART prescription (89.4% vs. 84.1%), retention in care (87.1% vs. 80.0%), recent viral suppression (77.2% vs. 70.9%), and sustained viral suppression (70.9% vs. 63.3%) compared to US-born adults, possibly due to greater Ryan White HIV/AIDS Program coverage and lower substance use and mental health burdens.	Adults with diagnosed HIV (US-born vs. non-US-born)

**Caruso et al., 2025. [19]**	Process and Outcome Evaluation of the Centers for Disease Control and Prevention's Think. Test. Treat TB Health Communications Campaign, United States.	Cultural stigma, limited social support, and psychological trauma contribute to delayed diagnosis and poor treatment adherence for HIV–TB co-infection among immigrant and refugee populations in the United States.	Mixed methods	To assess the CDC's Think. Test. Treat TB health communications campaign's reach, audience engagement, awareness, knowledge, perceptions, and behavioral intentions among non-US-born consumers and their health care providers.	Relied on self-reported survey data subject to bias, was localized to two metropolitan areas with electronic-device-owning participants, conducted shortly after rollout, limiting long-term assessment, and may have been confounded by concurrent TB interventions.	Leveraged robust digital and print media metrics alongside tailored, multilingual materials and standardized surveys to comprehensively measure both process and outcome indicators in the intended audience.	The campaign generated over 33 million impressions, 2066 warehouse orders, and high engagement, with 87.8% of exposed consumers intending to ask their provider about TB and nearly all providers finding materials relevant, though only 43.2% were likely to recommend LTBI testing to colleagues.	Non-US-born populations and healthcare providers

**Bitterfield et al., 2025 [22]**	Interventions to Improve Health Among Refugees in the United States: A Systematic Review.	Cultural misunderstandings, social marginalization, and psychological stressors such as trauma and fear of stigma hinder effective diagnosis and treatment of HIV–TB co-infection among immigrant and refugee populations in the United States.	Systematic review	To identify, characterize, and evaluate the effectiveness of patient-level healthcare interventions aimed at improving health outcomes among refugee populations in the United States.	Heterogeneity in intervention modalities, outcomes, and study designs precluded quantitative synthesis, evaluations often focused on short-term knowledge or satisfaction rather than long-term health or behavior change, and mental health interventions were excluded.	Utilization of a comprehensive, multi- database PRISMA search strategy and mixed- methods appraisal (MMAT) enabled inclusion of diverse study designs and rigorous quality assessment.	Interventions clustered into healthcare provision/management, resource provision, and education—TB received the most sustained, large-scale programs, whereas other health areas relied on piecemeal efforts, and most studies measured proximal outcomes like knowledge rather than concrete health improvements.	Refugee populations
**Tan et al., 2018 [9]**	Factors Associated with Health Insurance Status in an Asian American Population in New York	Cultural beliefs, social exclusion, and psychological trauma limit healthcare access and adherence to HIV–TB treatment among immigrant and refugee populations in the United States.	Quantitative	To identify factors associated with health insurance status among Asian American subgroups in New York City.	Non-generalizable due to convenience sampling, reliance on self-reported data, and cross-sectional design	Disaggregated analysis of underrepresented Asian subgroups and use of culturally sensitive, in-language data	Health insurance coverage varied significantly by subgroup and was influenced by factors like gender, length of U.S. residence, education level,	Asian American immigrants (NYC)

	City: Analysis of a Community-Based Survey.				that limits causal inference.	collection.	health status, and healthcare-seeking behavior.	

Author and Year	Title	Variables	Study Design	Purpose	Limitations	Strengths	Key Findings	Population
**Stryker et al., 2025 [17]**	Increasing Awareness About Tuberculosis Infection Screening in Latino Immigrants: A Community-Based Participatory Research Approach.	Cultural stigma, social isolation, and psychological distress create significant barriers to timely diagnosis and consistent treatment of HIV–TB co-infection among immigrant and refugee populations in the United States.	Mixed methods	To describe Latino immigrants' health beliefs about TB screening, co-design a culturally tailored educational intervention with community partners, and pilot its impact on screening intentions.	The small, predominantly female sample and cross-sectional pilot prevented measuring actual screening uptake and limited generalizability across genders and origin groups.	Partnering with a bilingual Community Advisory Board ensured intervention relevance, cultural appropriateness, and community ownership.	Viewing the co-created animated video significantly increased participants' perceived importance of TB screening (from 80% to 94%) and yielded high intention to be screened.	Latino immigrants

**Dhar et al., 2015 [8]**	Testing International Adoptees for Tuberculosis.	Cultural misperceptions about disease, social factors like second-hand smoke exposure and housing conditions, and psychological fears surrounding stigma and medical testing impact the diagnosis and management of HIV–TB co-infection among immigrant and refugee populations in the United States.	Quantitative study	To evaluate agreement and performance of IGRA versus TST for detecting latent tuberculosis infection in immigrant children.	Lack of a definitive gold standard for LTBI and potential BCG-related false positives complicate interpretation of discordant results.	Large, real-world pediatric cohort from TB-endemic regions enhances applicability to BCG- vaccinated populations.	TST and QFT showed poor concordance. TST-positive with only 17% QFT confirmation, and 2% QFT-positive despite negative TST—supporting greater reliance on IGRA.	Internationally adopted immigrant children
**Rocha-Jiménez et al., 2018. [2]**	Stigma and unmet sexual and reproductive health needs among international	Cultural stigma, social discrimination, and psychological trauma collectively hinder access to and engagement	Qualitative study	To explore migrant sex workers' experiences and narratives around unmet sexual and reproductive health needs and access barriers at the	Small, purposive sample of predominantly female participants limits	Ethnographic fieldwork across three border sites yielded rich, context- specific insights into structural and interpersonal	Stigma, legal-status constraints, cost barriers, and information gaps lead to significant unmet SRH needs and delayed or	

	migrant sex workers at the Mexico-Guatemala border.	with HIV–TB diagnosis and treatment services among immigrant and refugee populations in the United States.		Mexico–Guatemala border.	transferability and generalizability beyond the studied communities.	determinants of SRH access.	avoided care among migrant sex workers.	

**Author and Year**	**Title**	**Variables**	**Study Design**	**Purpose**	**Limitations**	**Strengths**	**Key Findings**	**Population**
Parmer et al., 2022. [23]	Latent tuberculosis infection: Misperceptions among non-U.S.-born-populations from countries where tuberculosis is common.	Cultural health beliefs, social instability, and psychological fear of deportation or discrimination reduce healthcare access and compromise HIV–TB diagnosis and treatment among immigrant and refugee populations in the United States.	Qualitative study	To identify misperceptions about latent TB infection and TB messages among non-US-born populations to guide culturally tailored communication.	Non-random, English fluent, higher-SES sample skews results and limits applicability to newly arrived or less educate immigrants.	Broad, diverse sample from six countries of origin and five high-incidence metropolitan areas ensured rich, context-specific insights.	Low LTBI awareness, confusion about BCG vaccine and TB testing, and concerns about stigma from targeted messages highlight the need for clear, stigma-reducing, audience-resonant LTBI communication strategies.	Non-US-born individuals from TB-endemic countries

**Oren. et al., 2016. [7]**	Detection of latent tuberculosis infection among migrant farmworkers along the US-Mexico border.	Cultural mistrust, social vulnerability, and psychological fear of stigma or legal consequences hinder effective HIV–TB diagnosis and treatment among immigrant and refugee populations in the United States.	Cross-sectional	To evaluate the compatibility and utility of TST versus QFT-GIT for detecting latent tuberculosis infection in this high-risk population and to identify demographic and clinical factors associated with test positivity.	Cross-sectional design, modest sample size with incomplete TST follow-up, missing BCG status for 40%, self-reported comorbidities, and limited generalizability beyond this regional cohort.	Real-world mobile clinic recruitment of a vulnerable, under-served group with paired TST and IGRA testing and comprehensive demographic and behavioral data.	QFT-GIT identified a higher LTBI prevalence (50%) than TST (34%), with fair concordance (κ = 0.38), suggesting reliance on IGRA may reduce under-diagnosis.	Migrant farmworkers
**Sheehan et al., 2018. [3]**	Role of Country of Birth, Testing Site, and Neighborhood Characteristics on Non linkage to HIV Care Among Latinos.	Cultural misconceptions, social barriers like language and poverty, and psychological stress from past trauma impede timely HIV–TB diagnosis and treatment among immigrant and refugee populations in	Quantitative study	To quantify disparities in linkage to HIV care by country/region of birth, testing site, and neighborhood characteristics.	Uses laboratory tests as a proxy for care (which may precede actual visits), lacks data on out-of-state or heritage-specific care, and may underestimate linkage if clients seek care	Leverages a large, population-based dataset with linked individual and ZCTA-level socioeconomic and demographic factors for robust, multilevel insights.	Higher non-linkage to care among those tested at blood banks and living in low-SES or low-Latino-density neighborhoods, while Cubans and Puerto Ricans were significantly more likely to link	Latino individuals with HIV

		the United States.			elsewhere.		within three months than US-born Latinos.	

Author and Year	Title	Variables	Study Design	Purpose	Limitations	Strengths	Key Findings	Population
**Vonasek et al., 2021. [26]**	Screening tests for active pulmonary tuberculosis in children.	Cultural stigma, limited social resources, and psychological trauma significantly affect access to and adherence with HIV–TB diagnosis and treatment among immigrant and refugee populations in the United States.	Quantitative study	To estimate the sensitivity and specificity of various screening tests for detecting active pulmonary tuberculosis in children at high risk (e.g., contacts, HIV-positive).	Incorporation bias from symptoms and CXR being part of the composite reference standard, heterogeneity in symptom definitions, and very limited data on Xpert MTB/RIF.	Rigorous methodology with dual independent data extraction, QUADAS-2 risk- of-bias assessment, and GRADE evaluation across diverse, real-world pediatric populations.	Symptom-based screens and CXR achieve moderate sensitivity (≈61–89%) and specificity (≈69–99%), but evidence for Xpert MTB/RIF is too sparse to draw firm conclusions, underscoring the need for better child TB screening tools.	

**Philbrick et al., 2025. [4]**	Pharmacist Interventions in Immigrant and Refugee Populations: A Scoping Review.	Cultural mistrust, social marginalization, and psychological fear of stigma and deportation hinder effective HIV–TB diagnosis and treatment among immigrant and refugee populations in the United States.	Scoping review	To identify and categorize pharmacist and student-pharmacist interventions addressing social determinants of health among immigrants and refugees.	Limited to 10 published articles (many abstracts excluded), small and heterogeneous interventions with no standardized outcome measures, constraining generalizability.	Comprehensive, librarian-assisted search across five databases with dual independent screening and rigorous SDOH mapping ensures breadth and methodological transparency.	Only 10 interventions were found, all targeting education access quality or health care access quality domains, leaving gaps in neighborhood built environment, social community context, and economic stability.	Immigrant and refugee populations
**Lin S. et al., 2016 [13]**	Systematic review of risk factors for nonadherence to TB treatment in immigrant populations.	Cultural stigma, social disadvantage, and psychological fear of discrimination negatively impact the diagnosis, treatment, and management of HIV–TB co-infection among immigrant and refugee populations in the United States.	Systematic review of 20 quantitative studies.	To synthesize and quantify which demographic, immigration-related, social, and clinical factors predict noncompletion of TB therapy among immigrants.	Marked heterogeneity in study designs, adherence definitions, risk-factor measurements, and confounder adjustment—plus restriction to English-language publications limits direct comparability	Comprehensive, multi-database search with dual screening, detailed critical appraisal, and inclusion of diverse geographic contexts ensure methodological rigor and broad applicability.	Undocumented immigration status, older age, and social vulnerabilities (e.g., homelessness, incarceration) were the most consistent predictors of TB treatment nonadherence.	Immigrant populations

					and generalizability.			
**Author and Year**	**Title**	**Variables**	**Study Design**	**Purpose**	**Limitations**	**Strengths**	**Key Findings**	**Population**
Bertelsen **et al., 2018. [18]**	Primary Care Screening Methods and Outcomes for Asylum Seekers in New York City.	Cultural misconceptions, social barriers like lack of insurance, and psychological distress such as fear and trauma hinder timely diagnosis and effective management of HIV–TB co-infection among immigrant and refugee populations in the United States.	Quantitative study	To evaluate primary care screening rates, disease prevalence, and follow up outcomes for infectious, non-communicable, and mental health conditions among asylum seekers.	Single-site, no external comparison group, limited to selected conditions and a unique torture-survivor population, restricting generalizability.	High real-world uptake through an interdisciplinary, culturally sensitive program with point-of-care testing and live interpreters, ensuring comprehensive data capture.	Very high burden of mental health and latent infection—71% screened positive for depression, 55% for PTSD, 41% for latent TB—while HIV (0.8%) and new hypertension (5.7%) detections were lower, underscoring critical screening needs.	Asylum seekers in NYC
